# Patient satisfaction in the spanish national health system: temporal trends and associated factors from 2018 to 2023

**DOI:** 10.1186/s12913-025-13649-x

**Published:** 2025-12-10

**Authors:** Francisco-Javier Prado-Galbarro, Montserrat Carmona-Rodríguez, Tasmania del Pino-Sedeño, Carlos Martín-Saborido, Carlos Sanchez-Piedra

**Affiliations:** 1 Departamento de Investigación, Hospital Infantil de México Federico Gómez, Ciudad de México, Mexico; 2 Agencia de Evaluación de Tecnologías Sanitarias, Instituto de Salud Carlos III, Madrid, Spain; 3 Red de Investigación en Cronicidad, Atención Primaria y Promoción de la Salud (RICAPPS), Madrid, Spain; 4 Servicio de Evaluación y Planificación del Servicio Canario de La Salud, Santa Cruz de Tenerife, Spain; 5 Fundación Canaria Instituto de Investigación Sanitaria de Canarias, Santa Cruz de Tenerife, Spain

**Keywords:** Patient satisfaction, Healthcare service, Public health, COVID-19, Social determinants of health

## Abstract

**Background:**

Patient satisfaction is a key indicator of healthcare system performance and legitimacy. Understanding its determinants provides valuable insights for policy design. To identify healthcare-related and socioeconomic determinants at both the individual and contextual levels (including regional indicators such as healthcare expenditure, physician density, life expectancy, and poverty rate) associated with satisfaction with the Spanish public healthcare system, and to assess changes in satisfaction levels before and after the COVID-19 pandemic.

**Methods:**

We conducted a cross-sectional analysis using nationally representative data from the Spanish Healthcare Barometer for the years 2018, 2019, 2022, and 2023. Individual-level data were linked with regional-level indicators, aggregated at the level of Spain’s autonomous communities, including public healthcare expenditure, physician density, life expectancy, and poverty rate. Descriptive statistics were used to examine temporal trends. For descriptive purposes, overall satisfaction was analyzed using the original 1–10 scale. For multivariate analysis, satisfaction was operationalized as a three-category ordinal outcome (low, moderate, and high satisfaction). Additional models included interaction terms with the pandemic period, defined as surveys conducted from March 2020 onwards, to assess changes in determinants over time.

**Results:**

The final sample included 29,146 adult respondents. Based on the original 1–10 scale, overall satisfaction declined significantly following the pandemic (from 6.66 to 6.26; *p* < 0.001), indicating a negative shift in public perception after COVID-19. Higher satisfaction was associated with better self-rated health, lower frequency of healthcare visits, and recent utilization of public healthcare services, including hospital, specialist, and emergency care. Sociodemographic factors such as being female, younger, and born outside of Spain were also positively associated with satisfaction. Lower satisfaction was observed among individuals with chronic conditions, lower socioeconomic status, or limited educational attainment. Contextual variables showed weaker associations, with only life expectancy exhibiting a significant positive relationship.

**Conclusions:**

Satisfaction with the Spanish healthcare system is primarily shaped by individual characteristics and experiences, with only limited evidence of associations with broader contextual indicators. The post-pandemic decline in satisfaction highlights the need for targeted policies that improve responsiveness, equity, and user confidence in public healthcare services.

## Introduction

Assessing the quality of healthcare systems is a fundamental aspect of public health research and policy planning. Patient satisfaction serves as a critical indicator of healthcare performance, influencing health outcomes, resource allocation, and system-wide improvements [1–3]. In Spain, the Healthcare Barometer provides valuable data on citizens’ perceptions of the healthcare system, offering an opportunity to analyze trends and identify factors associated with satisfaction levels [4].

Patient satisfaction, and not the satisfaction of healthcare professionals, is a multidimensional concept shaped by factors such as accessibility, waiting times, quality of medical care, interpersonal relationships with healthcare providers, and system efficiency [5]. Several studies have demonstrated that satisfaction is closely linked to the responsiveness of the healthcare system and the degree to which it meets patient expectations [6–8]. Understanding these factors can help policymakers design interventions that improve both patient experiences and overall system performance [9].

The COVID-19 pandemic significantly altered healthcare delivery, highlighting the importance of resilience and adaptability in health systems worldwide [10, 11]. In Spain, the pandemic placed extraordinary strain on hospitals, primary care, and emergency services, potentially affecting public satisfaction with the healthcare system [12].

Previous research indicates that demographic and socioeconomic variables, such as age, gender, education level, and income, play a crucial role in shaping satisfaction levels [5, 13]. Additionally, macroeconomic indicators, including regional healthcare expenditure per capita, may influence satisfaction by affecting service availability and quality [14]. In Spain, healthcare planning and provision are highly decentralized: while the central government sets overall legislation and financing rules, capacity planning and most healthcare expenditure decisions are executed at the level of the autonomous communities. This makes regional-level indicators, such as healthcare spending, directly relevant for understanding variations in satisfaction. The COVID-19 pandemic has further altered both individual experiences and system performance, reinforcing the need to examine how sociodemographic, socioeconomic and contextual determinants interact over time. Despite the existing literature on determinants of satisfaction, evidence for Spain remains limited with respect to recent temporal trends spanning the pre- and post-pandemic periods and the joint role of individual and regional indicators. This study addresses that gap using nationally representative Barometer data from 2018 to 2023 to provide integrated evidence on healthcare-related and social determinants of satisfaction with the Spanish public healthcare system. Multilevel analyses incorporating both individual and regional factors can provide a more comprehensive understanding of satisfaction drivers [15].

This study aims to assess changes in satisfaction with the Spanish healthcare system before and after the COVID-19 pandemic, identifying the determinants of variations in public perception based on data from the 2018, 2019, 2022, and 2023 waves of the Healthcare Barometer. By identifying key factors influencing satisfaction, policymakers can develop targeted strategies to increase trust and engagement with the healthcare system, ultimately improving population health outcomes.

## Methods

### Study design and data sources

We conducted a cross-sectional observational analysis using data from the Spanish Healthcare Barometer surveys (https://www.sanidad.gob.es/estadEstudios/estadisticas/BarometroSanitario/) for the years 2018, 2019, 2022, and 2023. The Healthcare Barometer is an annual survey commissioned by the Spanish Ministry of Health and implemented by the Centro de Investigaciones Sociológicas (CIS), which oversees sampling, data collection, and quality control. The authors of this study were not involved in survey design or fieldwork, and relied exclusively on anonymized public-use datasets. Each wave consists of an independent cross-sectional sample of residents aged 18 years and older; respondents are not followed longitudinally across years. These annual surveys are based on stratified random sampling and are administered via telephone interviews to adults residing in Spain. They collect perceptions and evaluations of the national healthcare system, along with a wide range of sociodemographic and health-related variables.

To incorporate contextual influences, we supplemented the survey data with regional-level indicators obtained from official and publicly accessible databases. Specifically, data on public healthcare expenditure per capita were retrieved from the Ministry of Health’s regional health accounts; physician density (number of physicians per 1,000 inhabitants) was sourced from the National Institute of Statistics (INE); life expectancy by sex and autonomous community was obtained from INE’s demographic and health statistics; and regional poverty risk rates were drawn from the Spanish Living Conditions Survey conducted by INE. All regional indicators were harmonized to match the corresponding year of the survey and aggregated at the level of Spain’s autonomous communities (Comunidades Autónomas).

### Study population

The study sample included all respondents from the selected survey years who provided data for the model’s variables. The response rate for complete surveys was exceptionally high across all study years, reaching 98.99% in 2018 (7,104 of 7,176), 99.37% in 2019 (7,111 of 7,156), 99.31% in 2022 (7,320 of 7,371), and 99.35% in 2023 (7,591 of 7,641). This high response rate minimizes the risk of non-response bias and validates the representativeness of our sample. Because the fraction of missing data was insignificant, the use of imputation methods was not considered necessary. The analysis was based on cases with available information for the key variables, which ensures the internal validity of the estimates and simplifies the interpretation of the results.

### Outcome variable

The dependent variable was satisfaction with the public healthcare system, assessed with the question: ‘Overall, how would you rate the functioning of the Spanish National Health System as a whole?’ (response scale 1 = very poor to 10 = very good). For descriptive analyses, we used the mean of this continuous scale. For regression analyses, responses were recoded into three categories: low (1–4), moderate (5–7), and high (8–10) satisfaction. This approach follows conventions in survey and health services research that enhance interpretability and comparability and is consistent with widely used practices such as “top-box” reporting in CAHPS surveys [16–18]. This dual reporting allows transparent presentation of temporal trends while ensuring interpretable categories for regression analyses.

### Independent variables

Explanatory variables included both individual-level and regional-level factors. At the individual level, we considered a set of sociodemographic characteristics, including age, sex, educational attainment, and social class. In addition, we included municipality size (< 10,000 inhabitants, 10,001–50,000 inhabitants, and > 50,000 inhabitants) and country of birth (Spain versus other). Health-related variables encompassed self-perceived general health status and whether the respondent had been hospitalized in the previous 12 months. A system-related factor included the self-reported waiting time for a consultation with a general practitioner in the public healthcare system (measured in calendar days and recorded in categorical bands as per the Barometer questionnaire), as well as the number of healthcare visits in the previous 12 months (reported per year).

At the regional level, we included contextual variables representing healthcare system characteristics and broader socioeconomic conditions. These comprised public healthcare expenditure per capita, the number of physicians per 1,000 inhabitants, life expectancy by sex, and the regional poverty risk rate. Indicators were aggregated at the level of Spain’s 17 autonomous communities and 2 autonomous cities (Ceuta and Melilla), which represent the first-order administrative divisions and the main units of healthcare financing and planning. All regional data were aligned to the corresponding year of the survey to ensure temporal consistency. For the main analyses, regional indicators were calculated as simple averages across autonomous community-years.

### Statistical analysis

We first conducted descriptive analyses to examine the distribution of key sociodemographic, health-related, and contextual variables across survey years, distinguishing between the pre-pandemic period (2018–2019) and the post-pandemic period (2022–2023). Weighted percentages with 95% confidence intervals (95% CI) and means and standard deviations (SD) were reported, accounting for the complex survey design.

To identify individual and contextual factors associated with healthcare system satisfaction, we fitted a multinomial logistic regression model, treating satisfaction as a categorical outcome with three levels: low (scores 1–4), moderate (scores 5–7), and high (scores 8–10). This modeling approach was selected after testing for the proportional odds assumption required for ordinal logistic regression, which was not met in our data. Prior to model estimation, we assessed multicollinearity among the predictors. The regional poverty rate showed a high Variance Inflation Factor (VIF), indicating collinearity with other socioeconomic variables. To preserve robustness and interpretability, this variable was excluded from the final model. Sensitivity analyses confirmed that its exclusion did not materially affect the results for other covariates. Models were estimated with the survey design (weights, strata, and primary sampling units). The Independence of Irrelevant Alternatives (IIA) assumption was assessed using the Small–Hsiao test; exploratory binary logistic models excluding the moderate category yielded stable and non-significant differences in coefficients, indicating that violations of the IIA assumption are unlikely to bias our findings. Results are presented as odds ratios (OR) with 95% CI, using low satisfaction as the reference category.

Selection of predictors for the multivariable models followed a hybrid approach. We first defined a set of variables a priori based on prior literature and theoretical relevance; these included demographic covariates (age, sex, country of birth), health-status indicators (self-rated health, chronic illness), socioeconomic measures (educational level, self-perceived social class), and healthcare utilization measures (number of healthcare visits per year, hospitalization in the past 12 months, use of emergency services, waiting time for PC consultation, and receipt of prescribed medication). Access to prescribed medicines was assessed with the item: “Inability to take the prescribed medication due to its economic cost.” Responses were “Yes,” “No,” or “Not prescribed.” For descriptive purposes, all three categories are reported. For regression analyses, a binary indicator was constructed (1 = Yes, 0 = No), excluding those who reported “Not prescribed,” as this group was not exposed to the risk of cost-related access barriers. Regional contextual indicators initially considered were public healthcare expenditure per capita, physician density and life expectancy; the regional poverty rate was excluded from the final multivariable model due to collinearity. In addition to these a priori variables, candidate predictors were screened in bivariate analyses and those showing a statistically significant association with the outcome (*p* < 0.05) were considered for inclusion in multivariable models. Age and sex were retained in all models irrespective of statistical significance. An additional model included interaction terms between selected predictors and a binary indicator for the pandemic period (defined as survey waves conducted from March 2020 onwards, corresponding to the declaration of the first state of alarm in Spain) to explore changes in associations over time. Goodness-of-fit was evaluated using the Akaike Information Criterion (AIC) and likelihood ratio tests.

All analyses were conducted using Stata statistical software version 16.0 (Stata, Stata Corp, College Station), with statistical significance set at *p* < 0.05.

### Ethical considerations

This study is based entirely on secondary, publicly available, and anonymized data. In accordance with applicable legal and regulatory frameworks (including the Spanish Organic Law 3/2018 on the Protection of Personal Data and Guarantee of Digital Rights) no formal ethical approval was required.

## Results

### Descriptive characteristics of the sample

The final analytical sample consisted of 29,146 adult respondents across four waves of the Spanish Healthcare Barometer (2018, 2019, 2022, 2023). The average age was approximately 50 years, with an even distribution by sex (51.5% female) and a predominance of individuals born in Spain (88.0%). A high proportion of respondents reported good or very good self-rated health (75.3%), while 39% indicated having a chronic condition. Educational attainment was relatively high, with 42.2% holding higher education degrees.

Average satisfaction with the healthcare system, measured on a scale from 1 to 10, was 6.46 (SD: 2.04). Descriptive statistics for key sociodemographic, health-related are summarized in Table 1A, and contextual characteristics are summarized in Table 1B.

**Table 1A:** Descriptive Characteristics of the Study Sample (Spanish Healthcare Barometer 2018–2023). *N* = 29,146 adult respondents. Respondent characteristics (Spanish healthcare barometer 2018–2023)

Variable	Reference category	Value (95% CI) / Mean (SD)
COVID period (%)	Before the pandemic	50.22 (49.59–50.85)
	During the pandemic	49.78 (49.15–50.41)
Overall satisfaction (1–10), mean (SD)		6.46 (2.04)
**Demographics**		
Age (years), mean (SD)		50.87 (17.71)
Sex (%)	Male	48.49 (47.87–49.12)
	Female	51.51 (50.88–52.13)
Country of birth (%)	Spain	87.99 (87.57–88.39)
	Other country	12.01 (11.61–12.43)
Municipality size (%)	≤ 10,000 inhabitants	20.75 (20.25–21.25)
	10,001–50,000 inhabitants	26.52 (25.97–27.07)
	> 50,000 inhabitants	52.74 (52.11–53.36)
**Self-perceived health**		
Self-rated health status (%)	Very Good	20.16 (19.66–20.67)
	Good	55.12 (54.49–55.74)
	Fair	20.45 (19.95–20.96)
	Poor	3.39 (3.17–3.62)
	Very por	0.89 (0.78–1.01)
Chronic illness (%)	Yes	38.86 (38.26–39.47)
**Socioeconomic characteristics**		
Educational level (%)	No education / Primary	14.07 (13.64–14.52)
	Secondary education	22.05 (21.53–22.58)
	Vocational training	21.28 (20.76–21.81)
	Higher education	42.24 (41.62–42.87)
	Other	0.35 (0.28–0.44)
Self-perceived social class (%)	Upper/upper-middle	12.88 (12.45–13.33)
	Middle	45.78 (45.14–46.42)
	Lower/lower-middle	41.34 (40.71–41.97)
**Healthcare utilization**		
Hospitalization in past year (%)	Yes	10.0 (9.68–10.42)
Emergency care use in past year (%)	Only public system	28.71 (28.15–29.28)
	Only private system	4.29 (4.04–4.55)
	Both systems	3.18 (2.96–3.41)
	No need for visits	63.82 (63.22–64.42)
Waiting time in PC (%)	Same day	11.75 (11.3–12.21)
	Next day	18.59 (18.04–19.15)
	More than one day	55.19 (54.47–55.9)
	More than one day, but attended on requested day	5.06 (4.75–5.4)
Inability to afford prescribed medication (%)	Yes	3.13 (2.91–3.36)
	No	89.43 (89.03–89.81)
	Not prescribed	7.45 (7.12–7.78)
Public hospital admission, mean (SD)		7.13 (2.01)
PC consultations, mean (SD)		6.8 (2.18)
Specialist consultations, mean (SD)		6.44 (2.22)
Public hospital emergency care, mean (SD)		6.14 (2.39)
PC emergency services, mean (SD)		6.48 (2.23)
Total number of healthcare visits, mean (SD)		0.83 (2.26)

Notes: PC = Primary Care. SD = Standard Deviation. Values are weighted percentages with 95% confidence intervals (CI 95%) or means with standard deviations, as appropriate. n refers to the unweighted sample count. Time unit for ‘Number of healthcare visits’ is per year. Waiting time for PC consultation is reported in calendar days based on survey categories. Ref: reference category. Educational level – Other: respondents whose qualifications did not fit into primary, secondary, or higher education. Medication access – Not prescribed: respondents who did not receive a prescription, and were therefore not exposed to cost-related access barriers

**Table 1B:** Descriptive Characteristics of the Study Sample (Spanish Healthcare Barometer 2018–2023). *N* = 29,146 adult respondents. Regional contextual indicators (Spanish healthcare barometer 2018–2023)

Variable	Mean (SD)
Demographics and Self-Perceived Health	
Life expectancy – total	83.33 (1.03)
Life expectancy – men	80.64 (0.99)
Life expectancy – women	85.92 (0.89)
Socioeconomic Characteristics	
Healthcare expenditure per capita (€)	1,596.82 (221.28)
Physician density (per 1,000 inhab.)	5.88 (0.80)
Nurse density (per 1,000 inhab.)	6.82 (1.08)
Regional poverty rate (%)	20.70 (7.26)

Notes: Contextual indicators are expressed as means with standard deviations, calculated across autonomous community-years

### Changes in participant characteristics and satisfaction before and after the pandemic

Table 2 presents a comparative analysis of sociodemographic characteristics, health status, healthcare utilization patterns, and contextual indicators before and after the onset of the COVID-19 pandemic. Statistically significant differences were observed across several key variables. Overall satisfaction with the healthcare system declined from a mean of 6.66 (SD: 1.86) before the pandemic to 6.26 (SD: 2.19) afterward (*p* < 0.001). A similar pattern was observed in user ratings for primary care, specialized care, and emergency services.

There was a notable increase in the proportion of respondents reporting chronic conditions (from 32.1% to 45.8%; *p* < 0.001), and a slight improvement in self-perceived health, with more individuals reporting “very good” health post-pandemic. Educational attainment and self-perceived social class also shifted markedly, with a substantial increase in the reporting of “other” educational levels post-pandemic, which likely reflects changes in survey categorization or reporting.

Healthcare utilization changed considerably. The proportion of individuals reporting hospital admissions, emergency visits (especially in the private sector or both public and private), and healthcare visits overall increased significantly after the pandemic. Additionally, waiting times for PC consultations lengthened, with fewer respondents being seen the same day or next day post-pandemic (*p* < 0.001). At the contextual level, per capita healthcare expenditure and physician density increased slightly, while regional poverty rates and life expectancy indicators remained relatively stable.

**Table 2:** Comparison of characteristics and satisfaction levels before and after the COVID-19 pandemic

Variable	Reference category	Pre-pandemic (*n* = 14,332)	Post-pandemic (*n* = 15,012)	*p*-value
Overall satisfaction (1–10), mean (SD)		6.66 (1.86)	6.26 (2.19)	< 0.001
**Demographics**				
Age (years), mean (SD)		49.8 (17.73)	50.13 (17.84)	0.158
Sex (%)	Male	48.48 (47.58–49.38)	48.51 (47.64–49.38)	0.964
	Female	51.52 (50.62–52.42)	51.49 (50.62–52.36)	
Country of birth (%)	Spain	88.7 (88.11–89.27)	87.27 (86.67–87.84)	< 0.001
	Other country	11.3 (10.73–11.89)	12.73 (12.16–13.33)	
Municipality size (%)	≤ 10,000 inhabitants	21.24 (20.54–21.96)	20.25 (19.56–20.96)	0.114
	10,001–50,000 inhabitants	26.56 (25.78–27.36)	26.47 (25.71–27.24)	
	> 50,000 inhabitants	52.20 (51.30–53.09)	53.28 (52.41–54.15)	
**Self-perceived health**				
Self-rated health status (%)	Very Good	17.74 (17.06–18.44)	22.6 (21.87–23.35)	< 0.001
	Good	57.46 (56.56–58.35)	52.76 (51.89–53.62)	
	Fair	20.54 (19.82–21.28)	20.36 (19.67–21.06)	
	Poor	3.6 (3.27–3.95)	3.18 (2.89–3.49)	
	Very por	0.67 (0.53–0.83)	1.1 (0.94–1.3)	
Chronic illness (%)	Yes	32.07 (31.23–32.91)	45.75 (44.89–46.62)	< 0.001
**Socioeconomic characteristics**				
Educational level (%)	No education / Primary	19.72 (19.01–20.46)	8.45 (7.98–8.94)	< 0.001
	Secondary education	27.49 (26.67–28.31)	16.64 (16–17.3)	
	Vocational training	19.44 (18.72–20.18)	23.11 (22.37–23.87)	
	Higher education	33.29 (32.43–34.16)	51.16 (50.28–52.04)	
	Other	0.06 (0.03–0.12)	0.65 (0.52–0.81)	
Self-perceived social class (%)	Upper/upper-middle	20.1 (19.36–20.85)	5.41 (5.01–5.84)	< 0.001
	Middle	36.33 (35.45–37.22)	55.56 (54.67–56.45)	
	Lower/lower-middle	43.57 (42.66–44.49)	39.03 (38.16–39.9)	
**Healthcare utilization**				
Hospitalization in past year (%)	Yes	8.9 (8.4–9.43)	11.19 (10.66–11.75)	< 0.001
Emergency care use in past year (%)	Only public system	25.25 (24.47–26.05)	32.19 (31.38–33.02)	< 0.001
	Only private system	2.79 (2.5–3.11)	5.8 (5.4–6.23)	
	Both systems	1.34 (1.14–1.57)	5.04 (4.66–5.44)	
	No need for visits	70.62 (69.79–71.44)	56.97 (56.1–57.83)	
Waiting time in PC (%)	Same day	16.02 (15.23–16.84)	8.63 (8.12–9.16)	< 0.001
	Next day	29.36 (28.35–30.4)	10.71 (10.17–11.29)	
	More than one day	48.9 (47.75–50.05)	59.78 (58.87–60.7)	
	More than one day, but attended on requested day	5.72 (5.19–6.3)	4.58 (4.2–4.99)	
Inability to afford prescribed medication (%)	Yes	2.8 (2.51–3.13)	3.45 (3.15–3.79)	< 0.001
	No	87.92 (87.32–88.5)	90.95 (90.43–91.44)	
	Not prescribed	9.28 (8.77–9.81)	5.6 (5.21–6.02)	
Public hospital admission, mean (SD)		7.06 (1.9)	7.21 (2.1)	< 0.001
PC consultations, mean (SD)		7.3 (1.8)	6.3 (2.41)	< 0.001
Specialist consultations, mean (SD)		6.82 (1.92)	6.07 (2.44)	< 0.001
Public hospital emergency care, mean (SD)		6.04 (2.25)	6.23 (2.52)	< 0.001
PC emergency services, mean (SD)		6.64 (2.03)	6.32 (2.41)	< 0.001
Total number of healthcare visits, mean (SD)		0.67 (2.15)	0.99 (2.37)	< 0.001

Notes: SD = Standard Deviation. PC: Primary Care. Educational level – Other: respondents whose qualifications did not fit into primary, secondary, or higher education. Medication access – Not prescribed: respondents who did not receive a prescription, and were therefore not exposed to cost-related access barriers

### Factors associated with satisfaction with the healthcare system

Table 3 presents sociodemographic characteristics, self-perceived health, socioeconomic status, and healthcare utilization according to levels of satisfaction with the healthcare system (low, moderate, and high). Respondents with high satisfaction were older on average (51.2 years vs. 48.3 years in the low group) and more frequently male. They were also more often born in Spain and resided in larger municipalities (> 50,000 inhabitants). In contrast, individuals with low satisfaction reported a higher prevalence of chronic illness (44.6%) compared to those with moderate (38.9%) or high satisfaction (37.3%).

Self-rated health status also varied significantly: those with high satisfaction more often described their health as “very good” (22.3%), whereas low satisfaction was associated with worse self-perceived health, including “poor” (6.3%) or “very poor” (2.5%).

Socioeconomic indicators showed a social gradient: respondents with high satisfaction were more likely to identify as upper or upper-middle class (14.2%) and slightly with greater level of education, while low satisfaction was more frequent among those with lower social class and primary education only.

Healthcare utilization patterns also differed. Those with high satisfaction reported more frequent use of hospital admissions, primary and specialist consultations, and both hospital and primary care emergency services. At the same time, waiting times for primary care appointments were shorter: 13.8% of the highly satisfied group obtained same-day care compared with 8.4% among the low satisfaction group.

Finally, inability to afford prescribed medication was more common in the low satisfaction group (6.2%) than in the high satisfaction group (2.2%).

**Table 3:** Sociodemographic, health, and healthcare utilization characteristics according to satisfaction level

Variable	Reference category	Low (*n* = 4,148)	Moderate (*n* = 8,455)	High (*n* = 16,553)	*p*-value
COVID period (%)	Before the pandemic	38.09 (36.48–39.72)	50.28 (49.12–51.45)	53.39 (52.56–54.23)	< 0.001
	During the pandemic	61.91 (60.28–63.52)	49.72 (48.55–50.88)	46.61 (45.77–47.44)	
**Demographics**					
Age (years), mean (SD)		48.31 (15.86)	48.26 (16.53)	51.18 (18.79)	< 0.001
Sex (%)	Male	46.73 (45.09–48.38)	45.46 (44.3–46.62)	50.57 (49.73–51.41)	< 0.001
	Female	53.27 (51.62–54.91)	54.54 (53.38–55.7)	49.43 (48.59–50.27)	
Country of birth (%)	Spain	92.52 (91.6–93.35)	90.7 (89.99–91.37)	85.32 (84.71–85.92)	< 0.001
	Other country	7.48 (6.65–8.4)	9.3 (8.63–10.01)	14.68 (14.08–15.29)	
Municipality size (%)	≤ 10,000 inhabitants	20.85 (19.56–22.20)	21.33 (20.40–22.28)	20.33 (19.68–21.00)	0.002
	10,001–50,000 inhabitants	28.26 (26.80–29.77)	27.04 (26.03–28.09)	25.83 (25.11–26.579	
	> 50,000 inhabitants	50.89 (49.25–52.53)	51.63 (50.46–52.79)	53.83 (53.00–54.67)	
**Self-perceived health**					
Self-rated health status (%)	Very Good	17.36 (16.14–18.66)	17.47 (16.6–18.39)	22.26 (21.57–22.97)	< 0.001
	Good	47.72 (46.08–49.37)	56.75 (55.59–57.9)	56.27 (55.44–57.1)	
	Fair	26.13 (24.71–27.59)	21.85 (20.91–22.82)	18.2 (17.57–18.86)	
	Poor	6.34 (5.58–7.19)	3.3 (2.91–3.73)	2.66 (2.41–2.95)	
	Very por	2.45 (2–3.01)	0.63 (0.48–0.84)	0.6 (0.48–0.75)	
Chronic illness (%)	Yes	44.64 (43.01–46.28)	38.95 (37.82–40.08)	37.28 (36.48–38.09)	< 0.001
**Socioeconomic characteristics**					
Educational level (%)	No education / Primary	10.94 (9.94–12.03)	12.3 (11.54–13.09)	15.78 (15.18–16.41)	< 0.001
	Secondary education	24.66 (23.23–26.13)	23.12 (22.15–24.13)	20.84 (20.16–21.55)	
	Vocational training	21.5 (20.15–22.91)	21.72 (20.77–22.71)	21.02 (20.33–21.73)	
	Higher education	42.51 (40.86–44.16)	42.61 (41.45–43.78)	41.95 (41.12–42.79)	
	Other	0.4 (0.23–0.69)	0.24 (0.15–0.38)	0.4 (0.31–0.52)	
Self-perceived social class (%)	Upper/upper-middle	9.29 (8.33–10.35)	12.2 (11.42–13.03)	14.18 (13.58–14.81)	< 0.001
	Middle	44.67 (43.00–46.35)	45.43 (44.25–46.61)	46.25 (45.39–47.11)	
	Lower/lower-middle	46.04 (44.36–47.73)	42.37 (41.20–43.55)	39.57 (38.73–40.41)	
**Healthcare utilization**					
Hospitalization in past year (%)	Yes	10.57 (9.6–11.62)	9 (8.36–9.68)	10.5 (10–11.02)	< 0.001
Emergency care use in past year (%)	Only public system	33.46 (31.92–35.05)	29.27 (28.22–30.35)	27.31 (26.57–28.07)	< 0.001
	Only private system	6.8 (6.01–7.68)	4.67 (4.2–5.19)	3.26 (2.97–3.58)	
	Both systems	6.53 (5.76–7.41)	3.69 (3.27–4.17)	2.04 (1.81–2.29)	
	No need for visits	53.2 (51.55–54.85)	62.36 (61.22–63.48)	67.38 (66.59–68.17)	
Waiting time in PC (%)	Same day	8.42 (7.44–9.52)	9.67 (8.91–10.48)	13.78 (13.14–14.45)	< 0.001
	Next day	11.47 (10.35–12.69)	16.81 (15.85–17.83)	21.56 (20.78–22.36)	
	More than one day	67.11 (65.34–68.83)	60.63 (59.32–61.94)	49.09 (48.12–50.07)	
	More than one day, but attended on requested day	2.92 (2.34–3.64)	4.48 (3.94–5.1)	6 (5.54–6.49)	
Inability to afford prescribed medication (%)	Yes	6.19 (5.42–7.06)	3.31 (2.91–3.76)	2.24 (2–2.5)	< 0.001
	No	87.17 (86.02–88.24)	89.29 (88.54–90)	90.26 (89.75–90.75)	
	Not prescribed	6.64 (5.87–7.5)	7.4 (6.8–8.04)	7.5 (7.07–7.96)	
Public hospital admission, mean (SD)		5.17 (2.39)	6.53 (1.7)	7.97 (1.49)	< 0.001
PC consultations, mean (SD)		4.68 (2.51)	6.2 (1.84)	7.68 (1.68)	< 0.001
Specialist consultations, mean (SD)		4.14 (2.33)	5.74 (1.81)	7.44 (1.73)	< 0.001
Public hospital emergency care, mean (SD)		3.98 (2.41)	5.39 (2.03)	7.13 (1.99)	< 0.001
PC emergency services, mean (SD)		4.39 (2.36)	5.83 (1.92)	7.41 (1.78)	< 0.001
Total number of healthcare visits, mean (SD)		1.32 (3.19)	0.85 (2.04)	0.69 (2.04)	< 0.001

Notes: SD = Standard Deviation. PC: Primary Care. Educational level – Other: respondents whose qualifications did not fit into primary, secondary, or higher education. Medication access – Not prescribed: respondents who did not receive a prescription, and were therefore not exposed to cost-related access barriers

The multivariate logistic regression analysis identified a range of individual and contextual variables significantly associated with reported satisfaction with the Spanish public healthcare system (Fig. 1A and 1B).

Among health-related variables, self-rated health showed a clear and strong gradient. Compared to respondents reporting “very good” health, the odds of high satisfaction were significantly lower for those with “fair” (OR = 0.69; 95% CI: 0.56–0.86), “poor” (OR = 0.50; 95% CI: 0.35–0.71), and especially “very poor” health (OR = 0.29; 95% CI: 0.15–0.56). A similar but less pronounced pattern was observed for moderate satisfaction, with lower odds for “poor” (OR = 0.65; 95% CI: 0.48–0.88) and “very poor” health (OR = 0.54; 95% CI: 0.32–0.90), while “fair” health was not significantly associated (OR = 1.01; 95% CI: 0.84–1.23). “Good” health was positively associated with moderate satisfaction (OR = 1.20; 95% CI: 1.03–1.39), though not with high satisfaction.

Sociodemographic variables were also important predictors. Respondents born outside Spain had higher odds of reporting high satisfaction (OR = 1.78; 95% CI: 1.42–2.24) and moderate satisfaction (OR = 1.34; 95% CI: 1.09–1.66). Female respondents were marginally more likely to report moderate satisfaction (OR = 1.15; 95% CI: 1.03–1.27), but not high satisfaction. Age was inversely associated with both moderate (OR = 0.99; 95% CI: 0.99–1.00; *p* = 0.005) and high satisfaction (OR = 0.99; 95% CI: 0.99–1.00), although the effect size was small.

Healthcare utilization patterns were strongly associated with satisfaction. Receiving care from public hospitals, primary care, specialist care, and emergency services were significantly associated with both high and moderate satisfaction. The odds of high satisfaction were highest for specialist care (OR = 1.63; 95% CI: 1.56–1.69), with a similarly positive association for moderate satisfaction (OR = 1.24; 95% CI: 1.20–1.28). Emergency services in primary care settings were also associated with moderate satisfaction (OR = 1.07; 95% CI: 1.03–1.10). A higher total number of visits was inversely associated with satisfaction in both categories (moderate: OR = 0.97; 95% CI: 0.95–0.99; high: OR = 0.95; 95% CI: 0.92–0.99).

Among contextual variables, life expectancy was only associated with high satisfaction (OR = 1.10; 95% CI: 1.01–1.19), but not with moderate satisfaction (OR = 1.0; 95% CI: 0.92–1.08). Individuals surveyed during the pandemic had lower odds of reporting satisfaction in both categories (moderate: OR = 0.82; 95% CI: 0.70–0.97; high: OR = 0.81; 95% CI: 0.68–0.97).

Finally, municipality size was positively associated with high satisfaction: respondents living in towns with 10,001 to 50,000 inhabitants (OR = 1.29; 95% CI: 1.09–1.52) and those in cities with more than 50,000 inhabitants (OR = 1.29; 95% CI: 1.11–1.50) reported higher satisfaction than those living in municipalities with fewer than 10,000 residents. No significant association was observed for moderate satisfaction.

**Fig. 1A:**
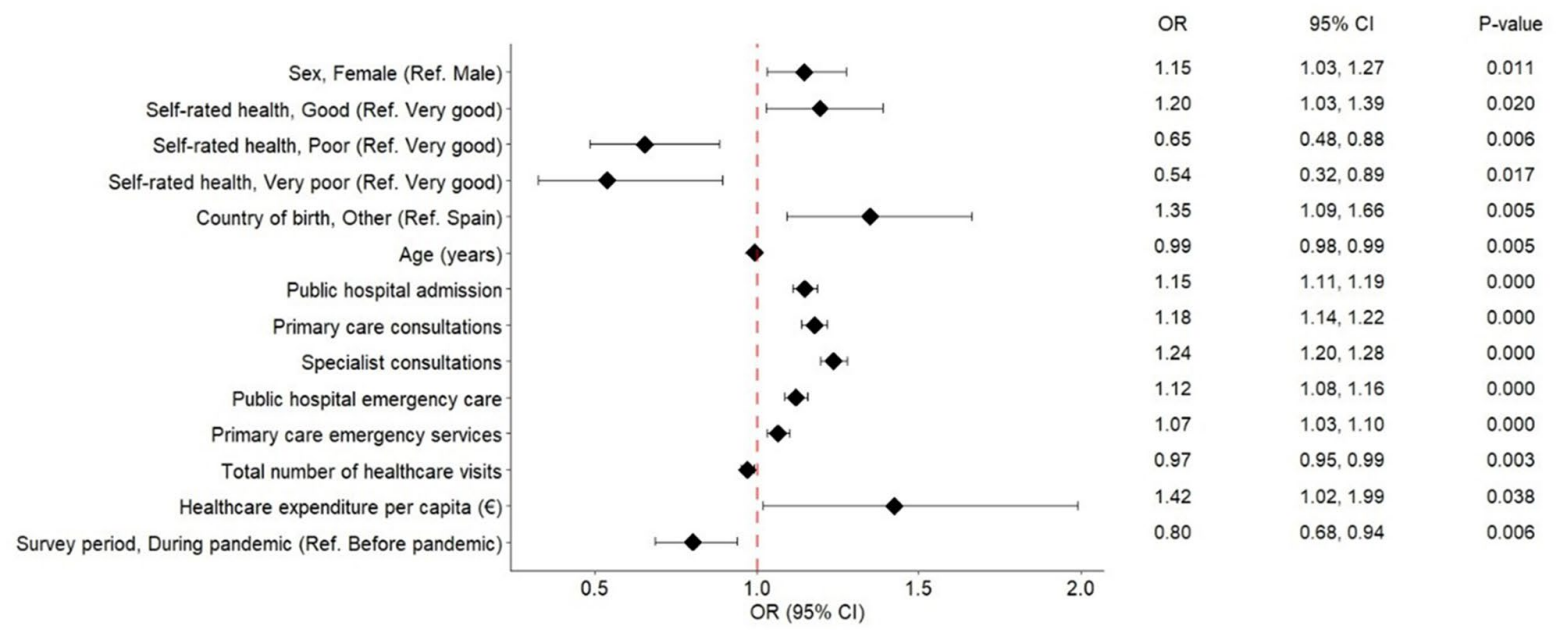
Adjusted Odds Ratios for Reporting Moderate Satisfaction With the Spanish Public Healthcare System (Reference: Low Satisfaction)

**Fig. 1B:**
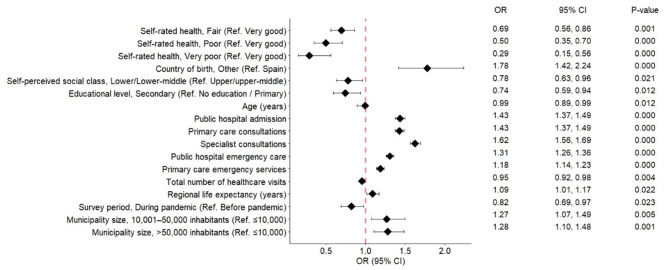
Adjusted Odds Ratios for Reporting High Satisfaction With the Spanish Public Healthcare System (Reference: Low Satisfaction)

Results from an multinomial logistic regression model using “low satisfaction” as the reference category. The outcome variable was a three-level satisfaction measure (low, moderate, high), based on responses to the general satisfaction question in the Spanish Healthcare Barometer (2018–2023). Odds ratios (OR) and 95% confidence intervals (CI) are presented for each factor. An OR > 1 indicates a higher likelihood of reporting high satisfaction compared to the reference category; values < 1 indicate a lower likelihood. The red dashed line denotes the null value (OR = 1.0).

## Discussion

This study provides a comprehensive analysis of individual and contextual factors associated with satisfaction with the Spanish public healthcare system, highlighting important shifts before and after the COVID-19 pandemic. Drawing on nationally representative data from four waves of the Spanish Healthcare Barometer, our findings reveal three key insights. First, overall satisfaction declined significantly in the post-pandemic period, reflecting broader changes in public perception and possibly unmet expectations during a period of heightened health system stress. Second, consistent with prior literature, satisfaction was strongly influenced by self-rated health, healthcare utilization, and sociodemographic characteristics such as education and social class. Notably, utilization of public healthcare services, particularly hospital care, specialist consultations, and emergency services, was associated with higher satisfaction, suggesting that contact with the system may reinforce trust and positive evaluations when access is successfully achieved. Third, contextual indicators such as healthcare expenditure and physician density showed limited explanatory power, underscoring the dominant role of individual experiences and expectations.

Beyond confirming the central role of individual characteristics, our findings offer a nuanced contribution to the existing literature on patient satisfaction. As highlighted by a recent study [19], patient satisfaction is increasingly recognized not only as an outcome indicator but also as a determinant of future healthcare utilization patterns and system sustainability. In this context, understanding what drives satisfaction is essential for anticipating economic implications and guiding efficient resource allocation. Consistent with prior evidence, self-perceived health emerged as one of the strongest predictors of satisfaction with the healthcare system. As reported in the systematic review by Batbaatar et al. [5], individuals with better health status are more likely to express higher satisfaction levels, reflecting both lower service needs and more favorable interactions with the system. Our study expands on this by showing a steep gradient in satisfaction across health categories, with individuals in “very poor” health being nearly 70% less likely to report high satisfaction, even after adjusting for healthcare utilization and socioeconomic status.

The positive association between satisfaction and recent contact with the healthcare system, particularly through public hospitals, specialist care, and emergency services, is consistent with international studies linking service use to favorable perceptions [20, 21]. However, we also observed that a higher frequency of healthcare visits was inversely associated with satisfaction, suggesting that while occasional or successful encounters may reinforce trust, frequent use might reflect unresolved health issues or dissatisfaction with continuity and coordination of care. This duality has been noted in prior analyses of U.S. data [22], where high utilization correlated with both higher expectations and more critical assessments of service delivery.

Socioeconomic gradients in satisfaction were also evident. Respondents with lower educational attainment and those identifying as belonging to lower social classes consistently reported lower satisfaction. This aligns with the findings of Xesfingi and Vozikis [13] and Djordjevic and Vasiljevic [23], who document the influence of structural determinants such as income, education, and perceived social status on health system evaluations. In the Spanish context, Valls Martínez and Ramírez-Orellana [8] similarly identified education and perceived income sufficiency as key determinants of satisfaction. These patterns underscore the importance of addressing equity and accessibility, not only in service provision but also in how users experience the healthcare system [3].

An intriguing finding in our study is the higher satisfaction reported by individuals born outside of Spain. This contrasts with some previous research where immigrant populations tend to report lower satisfaction due to barriers in access, communication, and cultural adaptation [24]. One possible explanation is that respondents’ comparisons with the health systems of their countries of origin may bias their evaluations in favor of the Spanish system, especially among individuals from countries with weaker or less accessible public healthcare infrastructures. Further research should explore these comparative dynamics in more detail.

Contrary to some expectations, contextual indicators such as physician density and regional healthcare expenditure per capita showed limited explanatory power in our model. While life expectancy at the regional level was positively associated with satisfaction, poverty rates and provider density did not exhibit statistically significant associations. This may reflect the fact that subjective satisfaction is more directly shaped by individual experiences than by macro-level system attributes, a conclusion echoed in the work of Vodă et al. [25], and supported by the multilevel comparative findings of Yuan [24].

Notably, some factors exhibited differential associations when comparing moderate and high satisfaction. For instance, respondents with “good” self-rated health were significantly more likely to report moderate satisfaction, but this association did not extend to high satisfaction levels. Similarly, variables such as educational attainment and perceived social class, which were clearly associated with high satisfaction, showed attenuated or non-significant effects in the moderate category. These patterns suggest that moderate satisfaction may reflect a distinct evaluative stance. Prior research has highlighted that moderate responses can capture uncertainty, conditional trust, or bounded expectations, particularly among users with limited exposure or lower perceived entitlement [26]. From a policy perspective, identifying what differentiates moderate from high satisfaction could inform targeted strategies to enhance user experience and build confidence in public services.

Finally, the decline in satisfaction observed during the post-pandemic period aligns with international trends indicating that the COVID-19 crisis disrupted both service delivery and public trust in health systems [11, 12]. In Spain, the reallocation of resources, delays in elective care, and primary care saturation likely contributed to the erosion of user satisfaction. This highlights the urgency of system recovery strategies focused not only on rebuilding capacity but also on restoring public confidence and managing expectations. These trends are consistent with pre-pandemic evidence from Spain showing that user satisfaction with the healthcare system was already experiencing fluctuations prior to COVID-19 [27], and that the pandemic may have accelerated or deepened existing perceptions of system fragility.

Our findings also suggest that satisfaction is not homogeneous across healthcare services. In emergency care, the immediacy of attention to urgent needs played a central role, and satisfaction remained relatively stable despite increased demand during the pandemic. In primary care, continuity and accessibility are key components for optimal functioning, and we observed longer waiting times after the pandemic, which negatively affected satisfaction. By contrast, specialist care is characterized by longer delays for consultations and diagnostic tests [28]. Thus, patient satisfaction at this level of care seems to depend more on overcoming access barriers than on the timeliness of service. These service-specific differences highlight that the type of health need (acute, chronic, or specialized evaluation) has distinct implications for satisfaction with the health system.

One of the main strengths of this study lies in the use of a large, nationally representative sample derived from the Spanish Healthcare Barometer, which offers high external validity and allows for the analysis of changes in public satisfaction over time and across population subgroups. The inclusion of both individual-level variables (such as self-rated health, healthcare utilization, and sociodemographics) and contextual indicators (such as regional healthcare expenditure and physician density) enabled a more nuanced understanding of the multilevel factors that shape citizens’ experiences with the healthcare system. In addition, the decision to examine the pre- and post-pandemic periods separately allowed us to capture temporal shifts in satisfaction, which is particularly relevant for health policy evaluation in the context of systemic shocks.

Our findings contribute to the international evidence base on factors associated with patient satisfaction, highlighting the importance of self-perceived health, socioeconomic status, and healthcare utilization patterns. From a policy perspective, these results underline the need for targeted strategies to reduce inequalities in satisfaction among individuals with poorer health and lower socioeconomic status, for example by reinforcing primary care and improving continuity of care. In addition, maintaining high levels of satisfaction among users of public hospitals and primary care services is crucial to sustain public trust in the National Health System. Finally, the observed decline in satisfaction during the post-pandemic period highlights the importance of resilience planning and resource allocation to strengthen the system’s capacity to respond to future health crises.

However, this study also has several limitations that should be acknowledged. First, the cross-sectional nature of the data limits our ability to establish causal relationships between explanatory variables and satisfaction levels. Second, although the logistic regression model accounts for a broad set of variables, unobserved confounders such as individual expectations, trust in institutions, or specific experiences with healthcare providers may still bias the estimated associations. Third, the outcome measure itself, satisfaction with the healthcare system, must be interpreted with caution. Satisfaction is a multidimensional and context-dependent concept influenced by expectations, norms, and previous experiences. While it is widely used in international health system assessments such as those conducted by the WHO, the OECD, or the Eurobarometer, it should be understood as an indicator of public perception rather than a comprehensive measure of system performance [29]. Fourth, the reliance on self-reported data introduces the possibility of recall and social desirability biases. Finally, although the survey records the exact country of birth, we analyzed this variable in binary form (born in Spain vs. outside Spain) to avoid sparse categories and unstable estimates. This approach inevitably masks heterogeneity across migrant groups, and future studies should explore differences in satisfaction by specific country or region of origin. In addition, our analytical decisions, including the recoding of the satisfaction scale into three categories and the classification of survey waves into pre- and post-pandemic periods, were made to enhance interpretability and comparability, but alternative specifications (for instance, different cut-off points or a finer temporal breakdown of pandemic phases) might yield different nuances in the findings. Moreover, although the Independence of Irrelevant Alternatives (IIA) assumption cannot be formally tested under complex survey design, exploratory analyses suggested that any potential violation is unlikely to bias the main conclusions. Despite these limitations, the study offers valuable insights into the dynamics of public satisfaction with healthcare in Spain and contributes evidence that can inform more responsive and equitable health system reforms. Future research may complement this approach through mixed-methods designs, combining survey data with qualitative studies to capture individual experiences and provide a more nuanced understanding of the determinants of satisfaction. In addition, future work should explicitly examine how different modalities of healthcare delivery, particularly the expansion of telemedicine and telephone consultations during and after the COVID-19 pandemic, influence satisfaction with the health system.

In conclusion, this study highlights how both individual experiences and broader systemic conditions contribute to shaping public satisfaction with the healthcare system in Spain. While sociodemographic and health-related characteristics remain important determinants, the pandemic context has intensified existing disparities and altered patterns of service use and evaluation. The observed decline in satisfaction post-COVID-19 underscores the need for targeted interventions to rebuild public confidence, particularly by improving accessibility and responsiveness across care settings. Monitoring satisfaction from a multidimensional and dynamic perspective remains essential to guide health policy in the post-pandemic era and to ensure that reforms resonate with citizens’ expectations and needs.

## Data Availability

The datasets analyzed during the current study are publicly available from the Spanish Ministry of Health’s official website: https://www.sanidad.gob.es/estadEstudios/estadisticas/BarometroSanitario/.

## Abbreviations

AIC: Akaike information criterion
CI: Confidence interval
COVID: Coronavirus disease
INE: Instituto nacional de estadística
MEPS: Medical expenditure panel survey
OR: Odds ratios
PC: Primary care
RICAPPS: Red de investigación en CroniCidad, atención primaria y promoción de la salud
SD: Standard deviation
